# Parathyroid carcinoma with sarcomatoid differentiation: a case report and literature review

**DOI:** 10.1186/s13000-020-01060-5

**Published:** 2020-12-14

**Authors:** Liang Hu, Xiaojun Xie

**Affiliations:** Department of Thyroid Surgery, The First Affiliated Hospital, Zhejiang University School of Medicine, No. 79 Qingchun Road, Hangzhou, 310003 PR China

**Keywords:** Parathyroid carcinoma, Sacroma, Parathyroid, Case report, thyroid, sarcomatoid differentiation

## Abstract

**Background:**

Parathyroid carcinoma (PC) is a rare thyroid tumor. PC with sarcomatoid differentiation(PCSD) is even rarer and its exact etiology remains unclear. We here report a case of PCSD, and present the clinicopathological features and pathological diagnosis and review the literature.

**Case presentation:**

A 71-year-old man presented with a mass of 4.5 cm × 3.5 cm in the right neck. The tumor was composed of nest-like transparent cells, and the septum had heterotypic rhabdoid cells with sarcomatoid differentiation. Immunophenotype was as follows: myogenic differentiation 1(MyoD1), myogenin and desmin were positive; clear cells were positive for chromogranin A(CGA), synaptophysin(Syn) and GATA-3; and Ki-67 proliferation index was 40%. Hematoxylin and eosin staining and immunohistochemistry were performed. The patient was diagnosed with PCSD, and died 6 months after surgery.

**Conclusions:**

PCSD is a rare type of primary parathyroid tumor with high malignancy and poor prognosis. Definitive diagnosis should be based on histopathological morphology and immunophenotype, and surgical treatment should be performed as soon as possible.

## Background

Parathyroid carcinoma(PC) is one of the rare cancers, accounting for less than 4% of cases of parathyroid diseases in the United States. DeQeurvain first described PC in 1904, which is characterized by high blood calcium and parathyroid hormone (PTH) levels [1]. However, PCSD is even rarer as a clinical solid tumor type. Nacamuli Randall first described this special type of parathyroid tumor in 2002 [2]. Since then, only four such cases have been reported including 2 cases abroad and 2 cases in China. The exact etiology of PC with sarcomatoid differentiation remains unclear. Typical clinical manifestations may include hypercalcemia and high PTH level. It does not differ significantly from a general PC, but the tumor is more aggressive and has poor prognosis.

## Case presentation

**Fig. 1:**
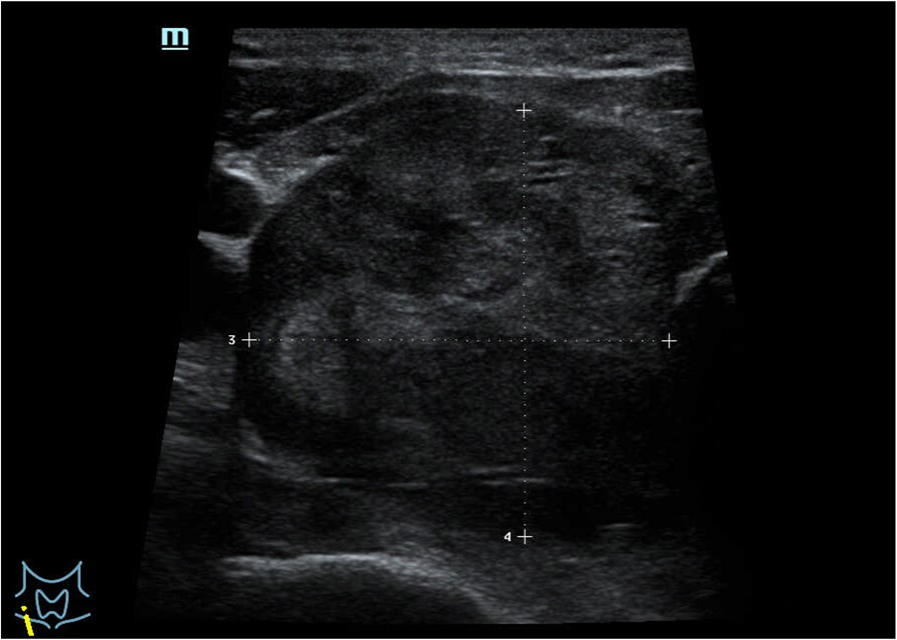
Ultrasonography showed a right thyroid mass

**Fig. 2:**
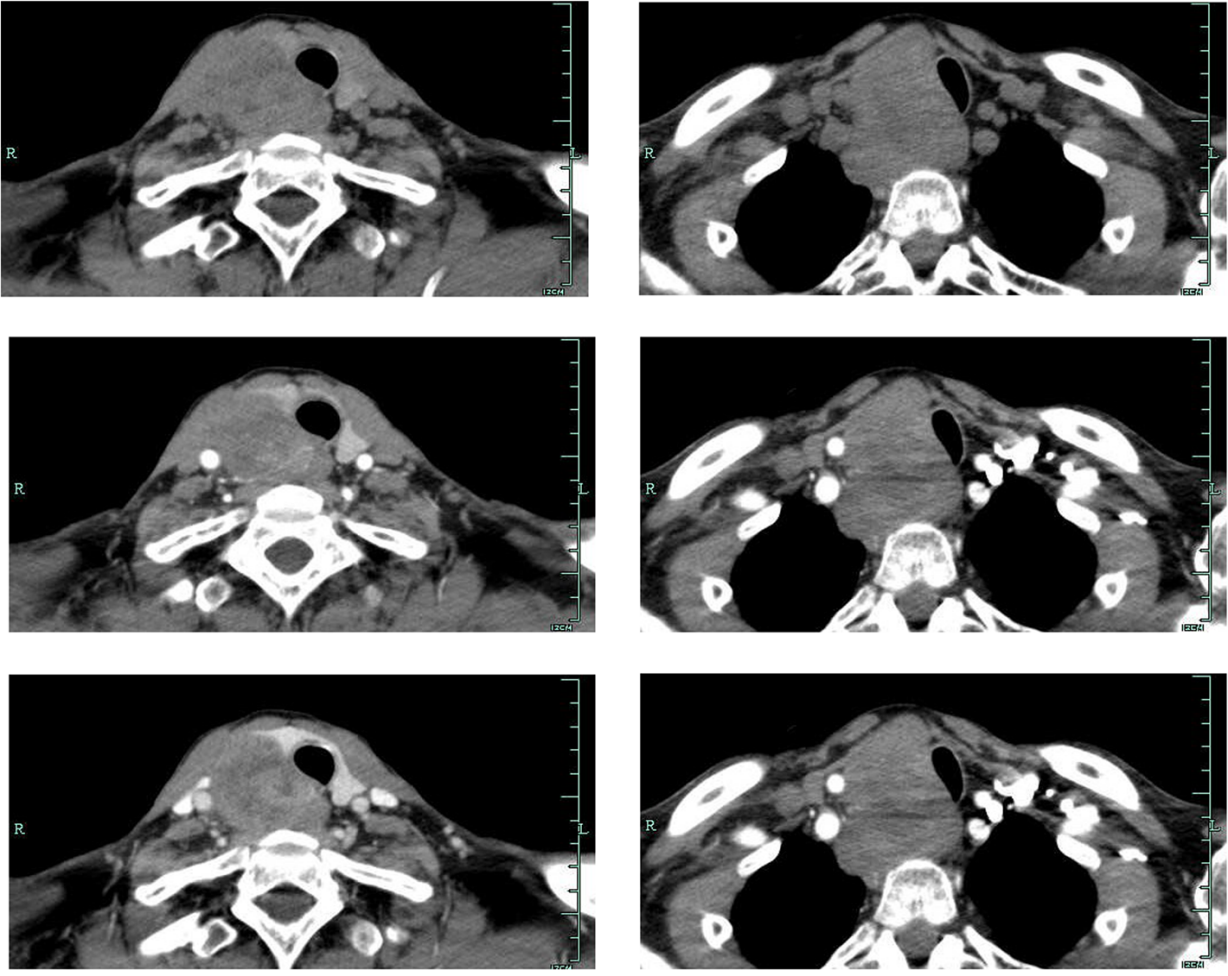
CT showed a large space-occupying lesion in the right thyroid region with invasion of the esophagus and mediastinum

**Fig. 3:**
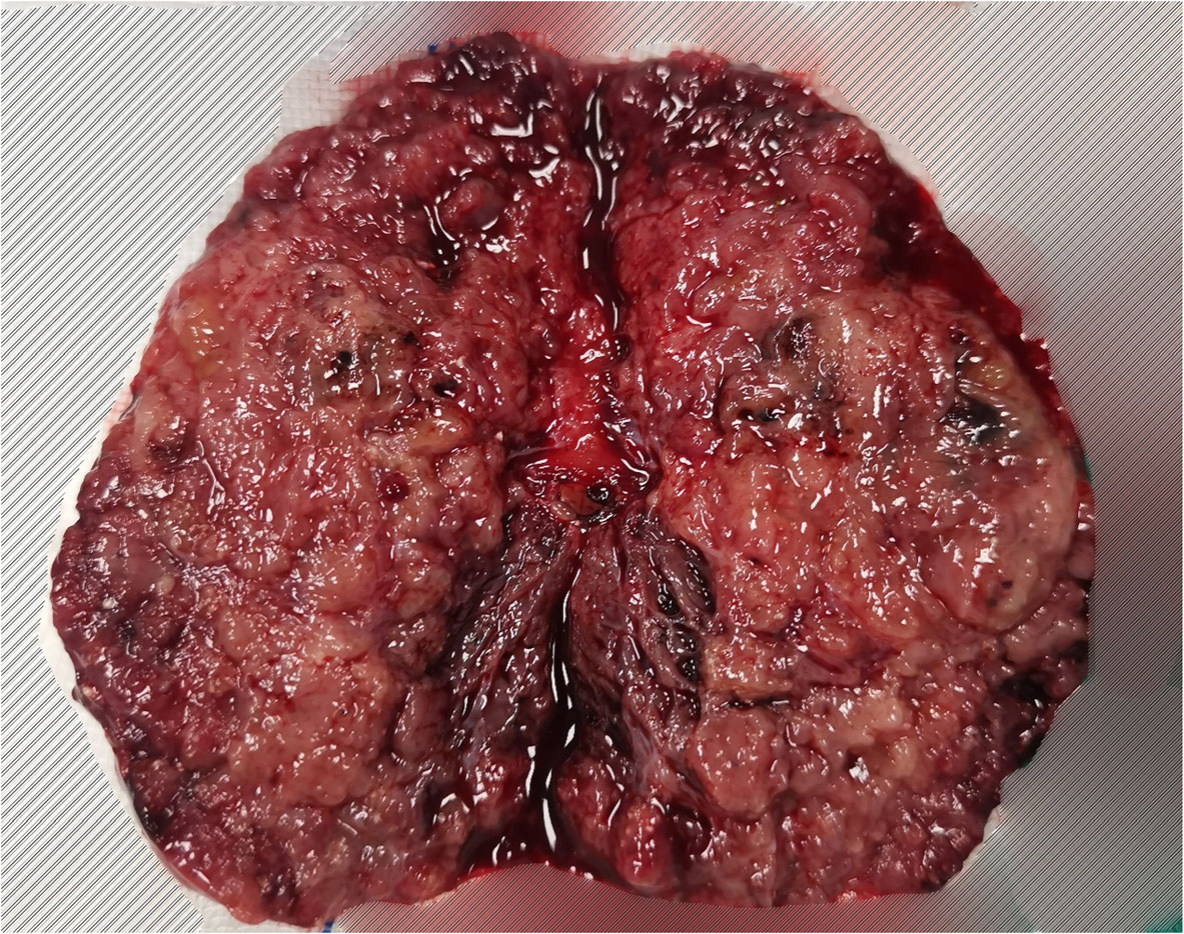
Gross image of the case

**Fig. 4:**
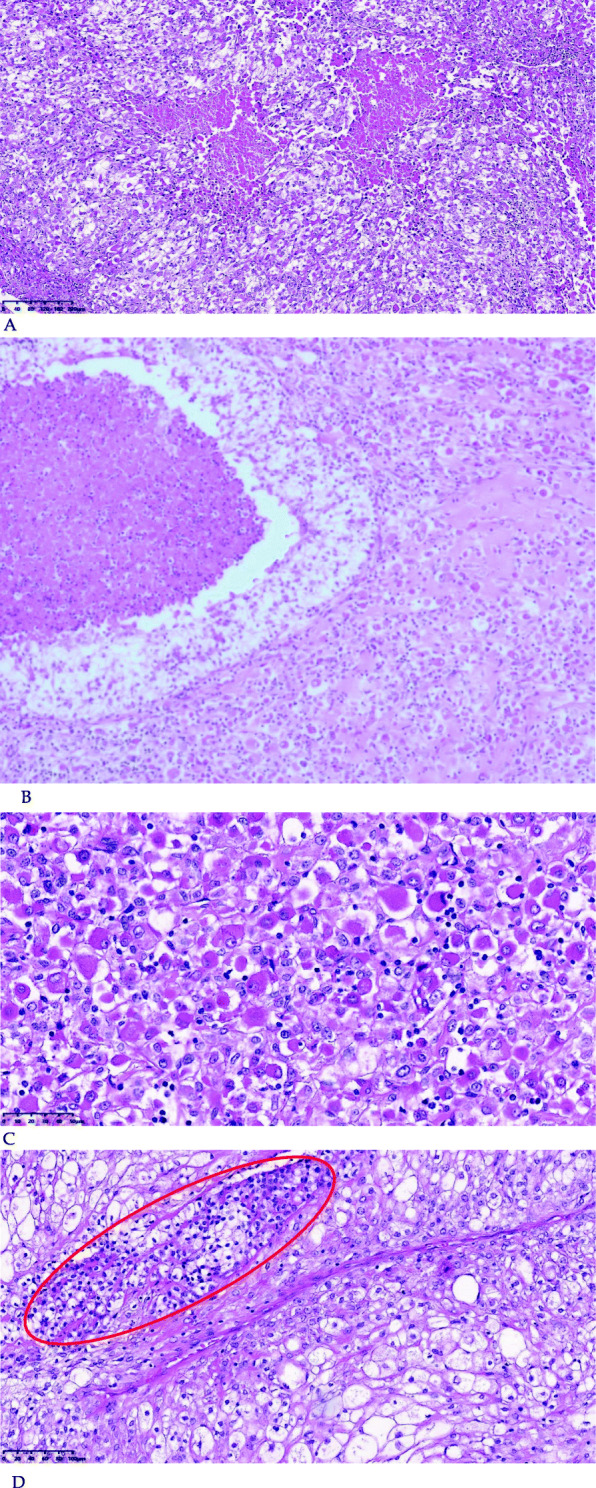
**a** Tumor cells with clear cytoplasm and rhabdomyoid tumor cells with eosinophilic nuclei intermingled with diffuse patchy arrangement and necrosis in the center [hematoxylin and eosin (HE) 100×]. **b** Junction between the typical parathyroid carcinoma cell region and the differentiated cell region of rhabdoid sarcoma (HE, 50×). **c** Rhabdoid tumor cells, eosinophilic cytoplasm, nuclear deviation, and obvious nucleolus (HE, 400×). **d** obvious characteristics of parathyroid glands: small cubic clear cells, abundant slender fibrovascular compartments (inside the red oval).(HE, 200×)

**Fig. 5:**
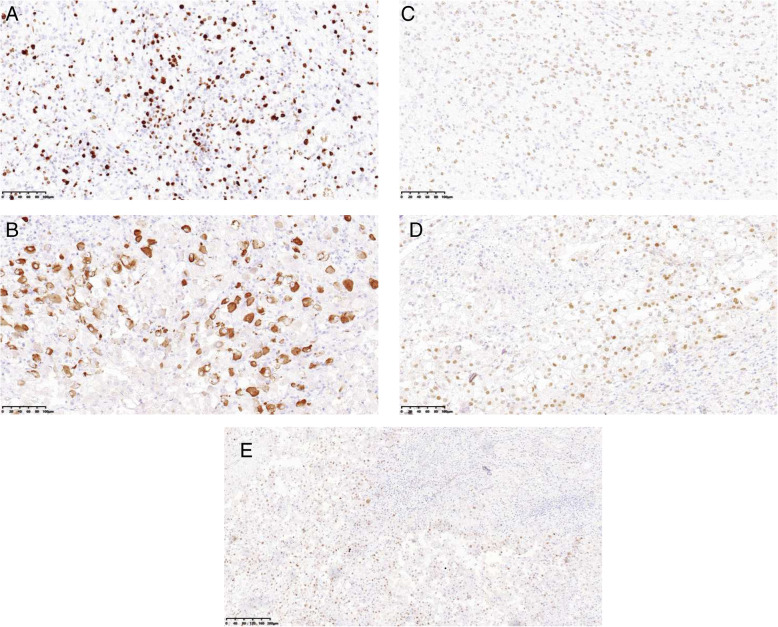
**a** Ki 67 proliferation index was about 40%(original magnification× 200). **b** Desmin partially positive (original magnification× 200). **c** GATA-3 positive (original magnification× 200). **d** MyoD1 partially positive (original magnification× 200). **e** myogenin partially positive (original magnification× 100)

A 71–year-old male patient was admitted to hospital for hoarseness for > 1 month. Ultrasound showed that the right thyroid was enlarged, bilateral thyroid nodules were present, the right larger nodules were about 4.5 × 3.5 cm, belonging to TI-RADS 4a type, and the left nodules belonged to TI-RADS 3 type (Fig. 1). Enhanced computed tomography (CT) showed a space-occupying lesion in the right thyroid area, invading the trachea and mediastinum (Fig. 2). Auxiliary examination showed that blood calcium was 2.34 (2.0–2.69) mmol/L, blood phosphorus 1.02 (0.87–1.45) mmol/L, PTH 89.1 (12.0–65.0) pg/ml, and tumor markers and other tests were all normal. Postoperative PTH was 40.9 (12.0–65.0) pg/ml, and serum calcium was 2.11 (2.0–2.69) mmol/L. Intraoperative exploration revealed a large mass of about 6 cm in the right thyroid area (Fig. 3), with unclear boundary, invading the esophagus and trachea,intraoperative frozen section pathology showed a malignant tumor with necrosis in the right thyroid area, which was confirmed by routine test and immunohistochemistry. Postoperative pathology suggested a malignant tumor in the right thyroid area, combined with immunohistochemical results, which was consistent with carcinosarcoma composed of rhabdomyosarcoma, and this case was of parathyroid origin (Fig. 4). Immunohistochemical results were as follows: cytokeratin CK5/6 (−), P63 (−),thyroglobulin(TG) (−), PAX8 (−), CK7 (−), CD 30(−), Ki-67(40%+++), Bcl-2 (−), cyclin D1 (+), HMB 45 (−), S-100 (−), melan A (−), transcription termination factor-1 (−), CK (Pan) (partial +), smooth muscle actin (−), desmin (partial +), MyoD1 (partial +), myogenin (partial +), epithelial membrane antigen (EMA) (partial +), CGA (partial +), Syn (partial +), TFE3 (−), GATA-3 (+), p53 (−) (Table 1 and Fig. 5). In this case, the capsule was thickened and parathyroid carcinoma cells were arranged in a diffuse sheet and trabecular manner. The tumor cells with clear cytoplasm and those with deviated eosinophilic nuclei were in a mixed, diffuse lamellar arrangement and central necrosis was seen. The tumor cells are large islands and sheets with foci of coagulative necrosis. Many cells with water-clear cytoplasm and sharp cell membrane, some of the cells are obviously eosinophilic, resembling rhabdomyoblasts, nuclei deviated, nucleoli are obvious, tumor cell nuclear division is not significant (Fig. 4d). The anaplastic thyroid carcinoma (undifferentiated carcinoma) usually has diverse morphology, obvious cell atypia, easy to see mitotic images, immunohistochemical PAX8 positive (36–76%), but this case has obvious clear cells, mitotic images are rare, PAX8 Negative, but GATA3 positive, which further proves that the tumor originates from the parathyroid gland. In addition to the classic microscopic features of PC, there were rhabdomyoid tumor cells with eosinophilic cytoplasm, nuclear deviation and obvious nucleoli (Fig. 4). During the operation, invasion of peripheral organs, elevated PTH, multiple positive immunohistochemical markers and genes were found, with rhabdomyosarcoma-like differentiation. After comprehensive consideration, Final diagnosis is parathyroid carcinoma with sarcomatoid differentiation(PCSD).

**Table 1:** antibodies list including clone and manufacturer in our case

Antibody	Result	Manufacturer	Product number	Clone
CK5/6	–	OriGene China	ZM-0313	OTI1C7
P63	–	Shanghai Long Island Antibody Diagnostica	M-0654	TP63/1786
TG	–	Shanghai Long Island Antibody Diagnostica	M-0495	SPM517
PAX-8	–	OriGene China	ZM0468	OTI6H8
CK7	–	Shanghai Long Island Antibody Diagnostica	0332	OV-TL12/30
CD30	–	OriGene China	ZM-0043	UMAB256
Ki-67	40%+++	OriGene China	ZM-0167	MIB1
Bcl-2	–	OriGene China	ZM0010	bcl-2/100/D5
Cyclin D1	+	OriGene China	ZA-0101	EP12
HMB45	–	OriGene China	ZM-0187	HMB45
S-100	–	OriGene China	ZA-0225	Rabbit polyclonal
Melan-A	–	Shanghai Long Island Antibody Diagnostica	0373	A103
TTF-1	–	OriGene China	ZM-0270	SPT24
CK(pan)	partially +	Shanghai Long Island Antibody Diagnostica	0349	AE1/AE3
SMA	–	OriGene China	ZM-0003	UMAB237
Desmin	partially +	OriGene China	ZA-0610	EP15
MyoD1	partially +	OriGene China	ZA0585	EP212
Myogenin	partially +	OriGene China	ZA-0592	EP162
EMA	partially +	OriGene China	ZM0095	UMAB57
CGA	partially +	Shanghai Long Island Antibody Diagnostica	0202	CGA/413
Syn	partially +	OriGene China	ZA-0506	EP158
TFE3	–	OriGene China	ZA-0657	EP285
GATA-3	+	OriGene China	ZM-0498	OTI5C11
P53	–	Shanghai Long Island Antibody Diagnostica	0430	SPM514

“-“represents negative,” +” represents positive

### Treatment and outcome

This patient underwent palliative resection of the right neck mass. Because the tumor invaded the surrounding organs severely and could not be completely separated, palliative resection was performed. This patient refused any further treatment after surgery, and died 6 months after surgery.

## Discussion and conclusions

PC is one of the rarest cancers. The 5-year survival rate of PC has been reported to be 78–85%, and the 10-year survival rate 49–77% [3–5]. It accounts for about 0.005% of all cancers [6]. The overall annual incidence rate is less than 1 case per million population [7, 8]. The Surveillance, Epidemiology, and End Results (SEER) database showed that the incidence rate of parathyroid carcinoma was 3.6/10 million in 2000–2012 [8]. The incidence rate of PC in Finland was 7.14/10 million from 2000 to 2013 [9]. According to Xing XP, a Chinese scholar, among patients with primary hyperparathyroidism (PHPT) confirmed by surgery and pathology, PC accounted for 3.10–10.53% [10], while PC accounted for < 1% of all PHPT patients in Europe and the United States, and 5% in Japan [11, 12].

The exact pathogenesis of PC remains unclear. At present, most researchers believe that the occurrence of PC is new rather than transformed from adenoma, which is based on the inference that there are different gene changes between parathyroid adenoma and adenocarcinoma. The major genes reported are cdc73/HRPT2 [13–15], gcm2 [16, 17] and prune2 [18]. The detection rate of cdc73/HRPT2 gene mutation in sporadic PC is 46–70% [19, 20]. Nonaka et al. considered that gcm2 is the main regulatory gene of parathyroid development, and the marker is only expressed in the parathyroid gland, including normal parathyroid tissue and all forms of benign and malignant parathyroid lesions [16]. Additionally, abnormal expression of noncoding RNA including miRNA and long noncoding (lnc) RNA may also be involved in the development of PC [21]. In the future, lncRNA PVT1, GLIS2-AS1 and anti-Gcm2 antibodies may become markers for the diagnosis of PC [22].

The diagnosis of PCSD is generally based on the combination of histology, biology and radiology. Multidisciplinary cooperation is the best model. The diagnostic standard is as strict as for thyroid follicular carcinoma. Capsule invasion and/or vascular invasion, perineural space infiltration, tumor perforation into surrounding tissues and/or metastasis should be present. The main criteria for diagnosis are as follows: (1) the cancer cells are arranged in trabecular shape with thick fibrous septum; (2) there is capsule or adjacent structure infiltration; (3) vascular invasion; (4) mitosis; (5) lymph node and/or other organ tissue metastasis; and (6) GATA3, cam5.2, SYN and CGA, which are important regulatory genes in parathyroid development, are positive. The loss of parafibromin and the high expression of PGP 9.5 and galectin-3 are helpful for the diagnosis of PC. At the same time, some tumor suppressor genes such as Rb, APC, p27 and BCL2 are often not expressed or weakly expressed. When Ki-67 index is > 5%, physicians should be alert to the possibility of malignant tumor [23].

Most PC patients have hypercalcemia, and about 3% of them have no clinical symptoms [24]. The results of biochemical tests and the diameter of parathyroid lesions in PHPT patients can predict PC. In PHPT, the best cut-off point for predicting the diameter of parathyroid lesions in PC is 3.0 cm [25]. A retrospective analysis showed that preoperative ultrasound examination of parathyroid lesions > 15 mm was valuable in the diagnosis of PC [26]. PCSD is rare and only five cases (including our case) have been reported in the literature (Table 2).

**Table 2:** Parathyroid carcinoma with sarcomatoid differentiation reported in the literature

Authors	Sex	Age (yr)	Maximum diameter mass (cm)	Blood calcium	Blood PTH	Positive Immunopheno type	Prognosis
Taggart et al.	F	57	4	Normal	Normal	CGA and vimentin were positive	Lung metastasis
Nacamuli et al.	M	54	9	Elevated	Elevated	AE-1, PTH, CGA, Syn, and desmin were positive	Lung metastasis, adrenal metastasis and death 7 mo after surgery
Zhang Haitao et al.	F	57	7	Elevated	Elevated	CK, Syn, PTH, Ki-67 was 50%	Unclear
Guan Zhongyan et al.	F	62	3.5	Normal	Undetermined	CK8/18, CGA, CD56, galectin-3 and vimentin were positive	Lung metastasis, and death 5 months after surgery
Present case	M	71	4.5	Normal	Elevated	Desmin, MyoD1, Myogenin, EMA, CGA, Syn, CK, GATA-3 were positive, Ki-67 was 40%	Esophageal and mediastinal invasion and death 6 mo after surgery

F represents female, M represents male

Among these five cases, there were more women than men, and the tumor diameter was > 3.5 cm, which was consistent with the report of Bae et al. The optimal cut-off point for predicting the diameter of parathyroid lesions was 3.0 cm. The serum calcium level of most patients with PC was significantly higher than 3.5 mmol/L. Serum PTH levels in patients with PC are usually 3–10 times higher than the upper limit of normal [25, 26]. Elevated serum calcium and PTH are more common in patients with PCSD. Therefore, when the serum calcium level is 3 mmol/L and the parathyroid lesion is > 3 cm (i.e., the so-called > 3 + > 3 rule) or ionic calcium > 1.77 mmol/L, physicians should be fully vigilant about the possibility of PC [27].

Radical resection is the only way to cure PCSD. The first operation is particularly important and should be performed as soon as possible. During the first operation for PC, parathyroid tumor with ipsilateral thyroid en bloc lobectomy including isthmus and ipsilateral central lymph node dissection should be performed [28–30]. If the tumor adheres to peripheral soft tissue, such as banded muscle and esophageal muscular layer, it should be removed as extensively as possible. If the recurrent laryngeal nerve is invaded, it should also be removed. Unfortunately, most of the PCSD have a high degree of malignancy. Most of them had distant metastasis in the early stage after surgery, and most of the patients died within 1 year after surgery.

Prophylactic lateral cervical lymph node dissection is generally not recommended because it does not prolong survival and may increase the incidence of complications. However, if lateral cervical lymph node metastasis is confirmed before surgery, therapeutic dissection is required. The biggest difficulty in the selection of surgical methods is the low accuracy of intraoperative frozen pathological diagnosis of PCSD. Unless there is obvious capsule, vascular invasion or regional lymph node metastasis, there are generally few direct reports of parathyroid cancer. When PCSD is diagnosed by parathyroid pathology after surgery, it is advisable to supplement surgery in time according to parathyroid cancer.

Chemotherapy drugs are generally ineffective against PCSD [31], and there are only a few successful reports [32]. PCSD is not sensitive to radiotherapy. Although there are reports of adjuvant radiotherapy to reduce local recurrence after the initial operation [33], due to the small number of cases and short follow-up time, adjuvant radiotherapy may only be used in PCSD patients with high risk of recurrence [34]. For local lesions, such as lung metastasis and vertebral metastasis, there are also individual cases of attempting radiofrequency ablation or absolute alcohol or combined percutaneous vertebroplasty to destroy metastases [35].

PCSD is a rare type of primary parathyroid tumor with high malignancy and poor prognosis. Definitive diagnosis should be based on histopathological morphology and immunophenotype, and surgical treatment should be performed as soon as possible.

## Data Availability

All data generated or analysed during this study are included in this published article.

## Abbreviations

PC: Parathyroid carcinoma
PCSD: Parathyroid carcinoma with sarcomatoid differentiation
PTH: Parathyroid hormone
CT: Computed tomography
PHPT: Primary hyperparathyroidism
CGA: Chromogranin A
Syn: Synaptophysin
TG: Thyroglobulin
MyoD1: Myogenic differentiation 1
